# A case of advanced systemic sclerosis with severe GERD successfully treated with acotiamide

**DOI:** 10.1186/s40792-016-0162-5

**Published:** 2016-04-13

**Authors:** Ryo Kato, Kiyokazu Nakajima, Tsuyoshi Takahashi, Yasuhiro Miyazaki, Tomoki Makino, Yukinori Kurokawa, Makoto Yamasaki, Shuji Takiguchi, Masaki Mori, Yuichiro Doki

**Affiliations:** Department of Gastroenterological Surgery, Graduate School of Medicine, Osaka University, 2-2, E-2, Yamadaoka, Suita, Osaka 565-0871 Japan; Division of Next Generation Endoscopic Intervention (Project ENGINE), Global Center for Medical Engineering and Informatics, Center of Medical Innovation and Translational Research, Osaka University, 2-2, Yamadaoka, Suita, Osaka 565-0871 Japan

**Keywords:** acotiamide, systemic sclerosis, gastroesophageal reflux disease (GERD), functional dyspepsia (FD), frequency scale for the symptoms of the GERD (FSSG)

## Abstract

The majority of systemic sclerosis (SSc) patients have gastrointestinal tract involvement, but therapies of prokinetic agents are usually unsatisfactory. Patients are often compromised by the use of steroid; therefore, a surgical indication including fundoplication has been controversial. There is no report that advanced SSc with severe gastroesophageal reflux disease (GERD) is successfully treated with acotiamide, which is the acetylcholinesterase (AChE) inhibitor designed for functional dyspepsia (FD). We report a 44-year-old woman of SSc with severe GERD successfully treated with acotiamide. She had received medical treatment in our hospital since 2003. She had been aware of the significant gastroesophageal reflux symptoms (e.g., heartburn, chest pain, and dysphagia) due to the development of esophageal hardening associated with SSc since 2014. As a result of upper gastrointestinal series, upper gastrointestinal endoscopy, and 24-h pH monitoring and frequency scale for the symptoms of the GERD (FSSG) scoring, she has been diagnosed with GERD associated with SSc. First of all, she started to take prokinetic agents Rikkunshito and mosapride and proton pump inhibitor; there was no change in reflux symptoms. So, we started to prescribe her the acotiamide.

After oral administration started, reflux symptoms have been improved. Five months after oral administration, FSSG score, a questionnaire for evaluation of the symptoms of GERD, was improved. Since its introduction of acotiamide, the patient has kept free from symptoms for 6 months.

## Background

Systemic sclerosis (SSc) is a multisystem and chronic disease characterized by abnormalities of small blood vessels and fibrosis of the skin and internal organs. SSc, when advanced, is often compromised with severe gastroesophageal reflux disease (GERD), which may be lethal in a worst-case scenario. A wide variety of medication has been used [1–5]; however, none of them are promising for patients with SSc. In addition, patients with SSc are often compromised by the use of steroid; therefore, a surgical indication including fundoplication has been controversial.

In this short communication, we describe our recent case of advanced SSc patients with severe GERD, who was successfully treated with a new drug originally designed for functional dyspepsia (FD).

## Case presentation

**Fig. 1:**
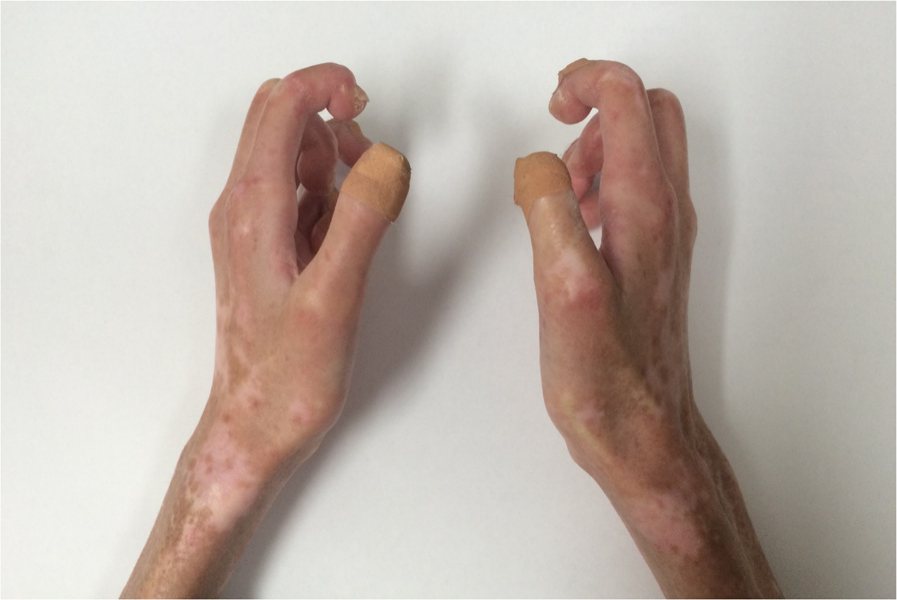
The abnormal build-up of fibrous tissue in the skin can cause the skin to tighten so severely that fingers curl and lose their mobility in SSc

**Fig. 2:**
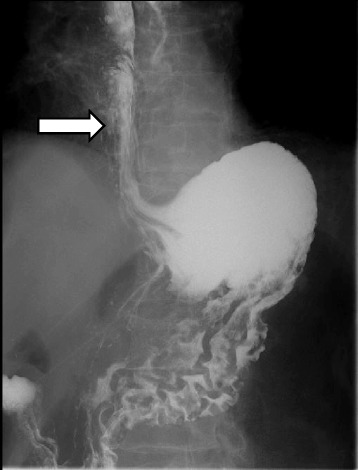
Upper gastrointestinal series revealed no expansion and meandering esophagus and reflux into the esophagus in the Trendelenburg position

**Fig. 3:**
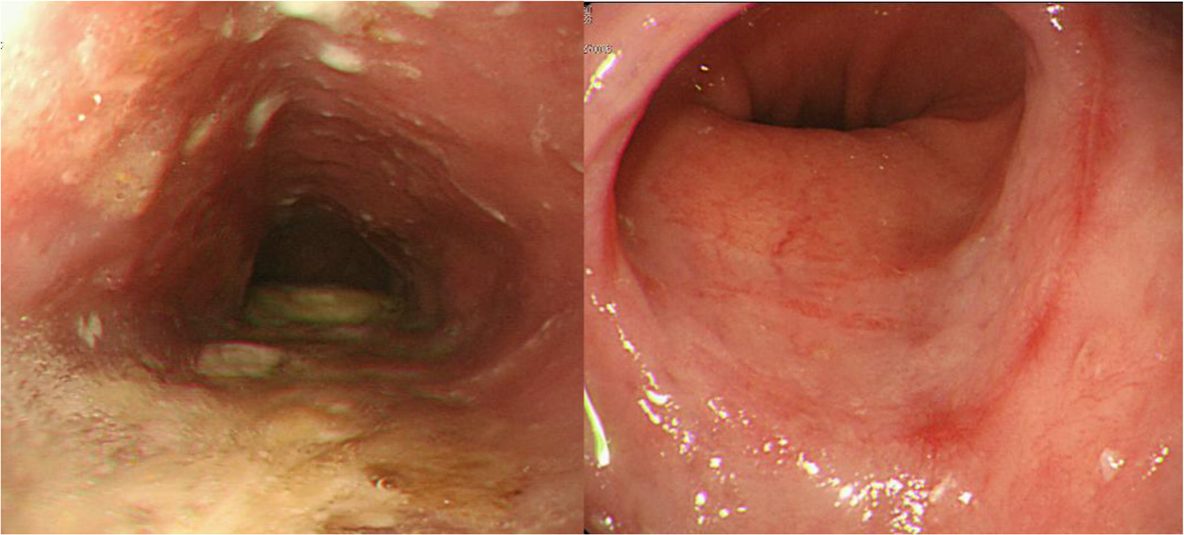
The upper gastrointestinal endoscopy showed reflux esophagitis of Los Angeles classification grade C and esophagus residue

A 44-year-old woman of SSc had received medical treatment in our hospital since 2003. She had been aware of the significant gastroesophageal reflux symptoms and esophagus stasis due to the development of esophageal hardening associated with SSc since 2014. On physical examination, cachexia, a “mouse face” appearance and ulceration in the distal phalanges were identified. The abnormal build-up of fibrous tissue in the skin can cause the skin to tighten so severely that her fingers curl and lose their mobility (Fig. 1). Because she had been aware of the worsening of gastroesophageal reflux symptoms, she received a medical examination from this department. Upper gastrointestinal series revealed no expansion and meandering esophagus, and reflux into the esophagus in the Trendelenburg position (Fig. 2). The upper gastrointestinal endoscopy showed reflux esophagitis of Los Angeles classification grade C and esophagus residue (Fig. 3).

**Fig. 4:**
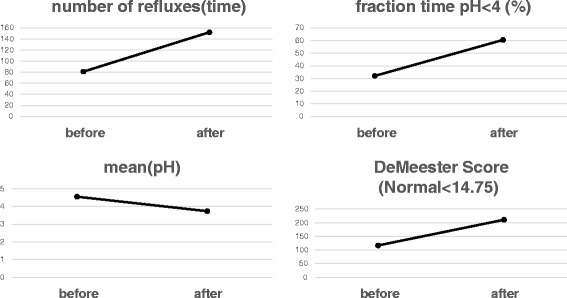
A 24-h pH monitoring was performed before and after acotiamide oral administration. These four items showed worsening before and after the administration

**Fig. 5:**
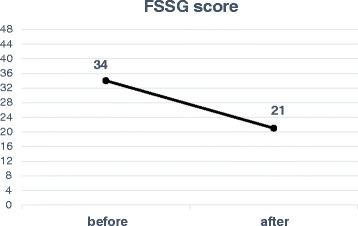
After oral administration of acotiamide, FSSG score was improved from 34 points to 21 points

A 24-h esophageal pH monitoring revealed significant acid reflux: the number of refluxes was 81 times, pH was below 4.0 for 32.1 %, mean pH was 4.55, and DeMeester score (normal <14.75) was 117.5 (Fig. 4). Symptoms of gastroesophageal reflux disease (frequency scale for the symptoms of the GERD (FSSG)) score, a questionnaire evaluating the symptoms of GERD, was 34 points [6] (maximum 48 points). As a result of these tests, she has been diagnosed with GERD associated with SSc. Treatment with Rikkunshito and mosapride, which are prokinetic agents, and proton pump inhibitor was started. However, her symptoms were not improved. Therefore, we started the acotiamide on June 2015, which was a new drug originally designed for FD. Since then, her symptoms which were heartburn, burp, and nausea after a meal were improved. Five months after acotiamide was started, the FSSG score was reduced to 21 points (Fig. 5). However, the results of 24-h esophageal pH monitoring showed worsening acid reflux: the number of refluxes was 152 times, pH was below 4.0 for 60.5 %, mean pH was 3.73, and DeMeester score (normal <14.75) was 211.6 (Fig. 4). The upper gastrointestinal series and upper gastrointestinal endoscopy did not change.

Since the introduction of acotiamide, the patient has been free from symptoms to date.

### Discussion

In 1994, Sjogren proposed a progression of SSc with gastrointestinal involvement, vascular damage, neurogenic impairment, and myogenic dysfunction with the replacement of normal smooth muscle by collagens fibrosis and atrophy [7]. It is distinguished in diffuse cutaneous SSc (dcSSc) or limited cutaneous SSc (lcSSc) whether skin hardening exceeds an elbow or a knee [8]. Next to the skin, the gastrointestinal tract is the second most common site of SSc organ damage that can affect patients with lcSSc and dcSSc [9]. It affects the gastrointestinal tract in more than 80 % of patients. Reflux esophagitis is found in 50–90 % of SSc patients [7, 10].

The common characteristics of GERD seen in SSc patients are as follows. The upper GI series often shows peristaltic decrease and expansion of the lower esophagus. The upper gastrointestinal endoscopy shows linear redness and erosion of the lower esophagus caused by the reflux, which often merges with the Barett esophagus. In addition, the merger frequency of esophageal cancer is often in SSc. Esophageal dysmotility leads to impaired acid clearance, and 24-h monitoring shows prolongation of esophageal exposure time to gastric acid [11].

Its treatment, either medical or surgical, has been still challenging. The major medical treatment option includes use of histamine-2 receptor antagonist (H2RA) and/or proton pomp inhibitor (PPI) for the purpose of controlling stimulation and inflammation of the esophageal mucosa by the gastric acid reflux [12–14]. Surgery, e.g., Nissen fundoplication, can be considered for drug-resistant reflux disease [15]. These medical/surgical treatments have been shown not as promising as those for reflux patients without SSc [16].

The last option for SSc patients with severe GERD is a group of prokinetic drugs. A wide variety of prokinetic agents have been used, such as mosapride citrate, metoclopramide, domperidone, erythromycin, octreotide, and dinoprost [1–5]. However, therapies of traditional prokinetic agents are usually unsatisfactory for severe GERD patients.

Acotiamide is the novel prokinetic agent basically designed for FD; it is the acetylcholinesterase (AChE) inhibitor. FD is a chronic disorder of sensation and movement (peristalsis) in the upper gastrointestinal tract. The acetylcholine (Ach) is released from cholinergic nerve terminals and lets the gastrointestinal tract shrink by binding to the muscarinic receptor of the gastrointestinal smooth muscle. It is thought that the Ach is broken down immediately by AChE and enterokinesis is regulated by this reaction. Acotiamide inhibits AChE and regulates the resolution of Ach. As a result, it increases the quantity of ACh available in the synaptic cleft and therefore improves enterokinesis [17].

We have prescribed a variety of traditional prokinetic agents, without obtaining even temporary relief of her symptoms. Therefore, we prescribed acotiamide. We performed upper gastrointestinal series, gastrointestinal endoscopy, 24-h pH monitoring, and the quality of life (QOL) scores for the assessment of gastroesophageal reflux before and after oral administration of acotiamide on this patient. No changes were observed before and after treatment in the upper GI series and upper gastrointestinal endoscopy. Acotiamide does not show the emission promoting effect on the normal gastric emptying in rats. However, prior research reports that acotiamide does improve the gastric emptying during restraint stress. Acotiamide might have led to symptom improvement due to the suppressed response to stress by decreasing the expression of NmU, a stress-related gene in the hypothalamus, via the vagus nerve [18].We think this may be a partial reason why our patient showed improvement of her GI symptoms.

In the 24-h pH monitoring, DeMeester score even showed a worsening from 117 to 211. However, FSSG, which is one of the established QOL scoring system, showed improvement from total 34 to 21 points. FSSG is a questionnaire composed of 12 questions and classified into two groups, which are five items related to “dysmotility” symptoms and seven items to “acid reflux” symptoms. In our case, the score related to both symptoms were improved from 17 to 10 points and 17 points to 11 points, respectively. As we describe later, these improvements can be explained by the pharmacological effects of acotiamide on both the gastric and the esophageal functions.

In fact, this patient was clearly aware of the improvement of clinical symptoms, and QOL has been improved.

In our case, traditional prokinetic agents attempted prior to acotiamide were not effective. Only acotiamide showed substantial relief of the patient’s symptom. We speculated this might be because of these two factors: (1) pharmacologically, acotiamide may not only affect the gastric emptying but also improve fundic accommodation of adaptive relaxation, and (2) acotiamide may directly act on the esophageal body and improve esophageal peristalsis. The authors have reached the above speculation based on the results of FSSG score: improvements of early satiety and chest discomfort. The question score “Do you feel full while eating meals?” was improved from 4 points to 2 points and “Do some things get stuck when you swallow?” was improved from 3 points to 2 points, respectively.

**Fig. 6:**
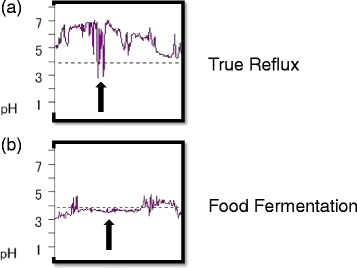
A 24-h pH tracing of this patient. True gastroesophageal reflux (**a**) and fermentation (**b**)

We just experienced one case; therefore, further accumulation of similar cases is definitely required. In our case, no objective improvement was observed on classical 24-h pH monitoring. The authors believe this examination might not be appropriate as an evaluation tool of GERD in patients with severe esophageal motor dysfunction like advanced SSc patients. Novais et al. reported that the 24-h abnormal pH tracings were classified into three types: (i) 24-h abnormal pH, with a true GERD pattern, i.e., sharp sudden pH drops, reaching values below 3 and then returning to usual esophageal pH (pH 6–7) (Fig. 6a); (ii) 24-h abnormal pH with a pattern suggesting esophageal fermentation due to retained food, i.e., steady drop of pH not reaching values below 3.0 (Fig. 6b); and (iii) negative 24-h pH, i.e., presence of physiological reflux (reflux episodes occurring in less than 4.5 % of total examining time) or zero reflux (absence of any episode of pH lower than 4.0) [19]. In a 24-h pH monitoring after acotiamide was started, there were many frequent waveforms of (ii) than those of (i). The esophageal food fermentation may have affected the results of this case. The QOL score, including FSSG is likely to accurately reflect the symptoms of patients than 24-h pH monitoring. Future tasks are to perform a detailed study by using a new method of measuring such as high resolution manometry.

## Conclusions

We have experienced a case of advanced SSc with severe GERD successfully treated with acotiamide. Acotiamide might become a help of advanced SSc with severe GERD patient whose surgical indication has been controversial.

## Consent

Written informed consent was obtained from the patient for publication of this case report and accompanying images. A copy of the written consent is available for review by the Editor-in-Chief of this journal.

## Abbreviations

ACh: acetylcholine
AChE: acetylcholinesterase
FD: functional dyspepsia
FSSG: frequency scale for the symptoms of the GERD
GERD: gastroesophageal reflux disease
SSc: systemic sclerosis
